# Synthesis of Carvedilol–Organotin Complexes and Their Effects on Reducing Photodegradation of Poly(Vinyl Chloride)

**DOI:** 10.3390/polym13040500

**Published:** 2021-02-06

**Authors:** Omar G. Mousa, Gamal A. El‐Hiti, Mohammed A. Baashen, Muna Bufaroosha, Ahmed Ahmed, Ahmed A. Ahmed, Dina S. Ahmed, Emad Yousif

**Affiliations:** 1 Department of Chemistry, College of Science, Al-Nahrain University, Baghdad 64021, Iraq; omarchemistry1987@gmail.com (O.G.M.); ahmedahmedalazawi@gmail.com (A.A.A.); emad_yousif@hotmail.com (E.Y.); 2 Cornea Research Chair, Department of Optometry, College of Applied Medical Sciences, King Saud University, P.O. Box 10219, Riyadh 11433, Saudi Arabia; 3 Department of Chemistry, College of Science and Humanities, Shaqra University, Dawadmi 11911, Saudi Arabia; mbaashen@su.edu.sa; 4 Department of Chemistry, College of Science, United Arab Emirates University, P.O. Box 15551, Al-Ain 1818, United Arab Emirates; muna.bufaroosha@uaeu.ac.ae; 5 Polymer Research Unit, College of Science, Al-Mustansiriyah University, Baghdad 10052, Iraq; drahmed625@gmail.com; 6 Department of Medical Instrumentation Engineering, Al-Mansour University College, Baghdad 10067, Iraq; dinasaadi86@gmail.com

**Keywords:** carvedilol–tin complexes, poly(vinyl chloride), photostabilizers, surface morphology, weight loss, functional group indices

## Abstract

Poly(vinyl chloride) (PVC) undergoes photodegradation induced by ultraviolet (UV) irradiation; therefore, for outdoor applications, its photostability should be enhanced through the use of additives. Several carvedilol tin complexes were synthesized, characterized and mixed with PVC to produce thin films. These films were irradiated at 25 °C with a UV light (λ = 313 nm) for up to 300 h. The reduction in weight and changes in chemical structure and surface morphology of the PVC films were monitored. The films containing synthesized complexes showed less undesirable changes than the pure PVC film. Organotin with a high content of aromatics was particularly efficient in inhibiting photodegradation of PVC. The carvedilol tin complexes both absorbed UV light and scavenged radicals, hydrochloride, and peroxides and, therefore, photostabilized PVC.

## 1. Introduction

Poly(vinyl chloride) (PVC) has numerous industrial applications [1]. It is one of the three most manufactured thermoplastics [2]. PVC has received considerable attention due to its low cost, long duration, good mechanical and chemical stability, ease of molding, and ability to be produced in different forms (rigid and flexible) [3,4]. It is widely used for the production of items such as medical equipment, toys, construction materials, food packaging, and disposable blood bags [2]. It is considered to be an excellent alternative to glass and wood in many applications [5].

PVC undergoes autocatalytic dehydrochlorination when exposed to a high temperature. This results in the formation of conjugated double bonds because of the elimination of hydrogen chloride (HCl) [6]. Therefore, the photostability of PVC is poor, and it must be mixed with appropriate photostabilizers to inhibit its decomposition and photooxidation. Various types of thermal stabilizers are used for PVC [7]. Recent research has focused on the production and use of organic PVC photostabilizers that are inexpensive to produce, can be used at low concentrations, and are non-toxic. Various types of small molecules are mixed with PVC to serve as photostabilizers and photoabsorbers. Such molecules can protect PVC from surface cracking, discoloration, and changes in mechanical properties under long periods of exposure to sunlight [8,9,10].

Recently, various additives, including polyphosphates [9,10], aromatics [11,12], Schiff bases [11,12,13,14,15,16,17,18,19], pigments [20], inorganic salts [21,22], and organotin complexes [23,24,25,26,27,28,29] have been tested for their efficiency as inhibitors of PVC photodegradation and photooxidation. Organotin complexes are highly stable, have distinctive properties, and are useful materials in numerous applications [30,31]. Carvedilol is available commercially, it is inexpensive, ecofriendly, non-toxic, and highly aromatic, and it contains heteroatoms (nitrogen and oxygen). These properties make carvedilol a good ligand for coordination with tin to produce effective PVC additives. This study reports the synthesis and characterization of new carvedilol tin complexes. These complexes are non-volatile, cause no change in color, are compatible with polymeric materials, can be used at a low concentration, and have been tested as inhibitors of PVC photodegradation under exposure to UV light.

## 2. Materials and Methods

### 2.1. General

Triphenyltin chloride (95%; Ph_3_SnCl), dibutyltin oxide (98%; Bu_2_SnO), dibutyltin dichloride (96%; Bu_2_SnCl_2_), dimethyltin dichloride (97%; Me_2_SnCl_2_), carvedilol (98%), and solvent were supplied by Merck (Gillingham, UK). The PVC (${\bar{M}}_{V}$ = *ca*. 250,000) was obtained from Petkim Petrokimya (Istanbul, Turkey). The FTIR spectra were recorded on a Shimadzu FTIR 8300 spectrophotometer(Shimadzu, Tokyo, Japan). The ^1^H (500 MHz), ^13^C (125 MHz), and ^119^Sn NMR (149 MHz) spectra were recorded on a Bruker DRX500 MHz spectrometer. The conductivity was measured on a WTW ProfiLine Oxi 3205 conventional portable meter in dimethyl sulfoxide (10^−3^ mole/L) at 25 °C (Geotech, Barcelona, Spain). The PVC optical images were captured using a Meiji Techno Microscope (Tokyo, Japan). A Veeco system was used to examine the surface morphology of PVC films. An accelerated weather-meter QUV tester was used to irradiate the PVC films using UV light with λ_max_ of 365 nm and a light intensity of 6.43 × 10^−9^ ein dm^−3^s^−1^.

### 2.2. Synthesis of Organotin Complexes **1** and **2**

A mixture of carvedilol (0.41 g, 1.0 mmol) and either triphenyltin chloride (Ph_3_SnCl; 0.39 g, 1.0 mmol) or dibutyltin oxide (Bu_2_SnO; 0.25 g, 1.0 mmol) in methanol (MeOH, 15 mL) was refluxed either for six or eight hours, respectively. The solvent was removed under vacuum and the solid produced was recrystallized from MeOH to yield a white powder of either of the organotin complexes **1** or **2**.

### 2.3. Synthesis of Organotin Complexes **3** and **4**

A mixture of carvedilol (0.81 g, 2.0 mmol) and dialkyltin chloride (1.0 mmol) in MeOH (20 mL) was refluxed for eight hours. The solvent was removed under reduced pressure and the solid produced was recrystallized from MeOH to yield a white powder of either of the organotin complexes **3** or **4**.

### 2.4. Preparation of PVC Films

Organotin complexes (25 mg) were added to a stirred solution of PVC (5 g) in tetrahydrofuran (THF; 100 mL). The mixture was stirred for three hours at 25 °C. The homogenous solution obtained was transferred onto glass plates containing holes (thickness = 40 μm) and left to dry at room temperature for 24 h and subsequently left at reduced pressure for eight hours.

### 2.5. Characterization of PVC Photodegradation Using FTIR Spectrophotometry

The progress of the photodegradation of the PVC was monitored using FTIR spectroscopy. The increases in the absorption of several functional groups as a function of irradiation time were recorded for the PVC films. The indices of functional groups (*I*s) were calculated using Equation (1) based on the absorbance of the peaks of corresponding functional groups (*A*s) and that of a reference group (*Ar*) that is not affected by irradiation (CH_2_) [32].

(1) $$I_{s}\;=\;A_{s}/A_{r}$$

### 2.6. Monitoring of PVC Photodegradation Using Weight Loss

PVC photodegradation results in the elimination of volatiles and, therefore, weight loss. The percentage of weight loss can be calculated from the weight of PVC before (*W*_1_) and after irradiation (*W*_2_) using Equation (2) [27].

(2) $$Weight\;loss\;\left(\%\right)\;=\;\left[\left(W_{1}-W_{2}\right)/W_{0}\right]\;\times \;100$$

## 3. Results and Discussion

### 3.1. Synthesis of Tin Complexes **1–4**

**Scheme 1:**
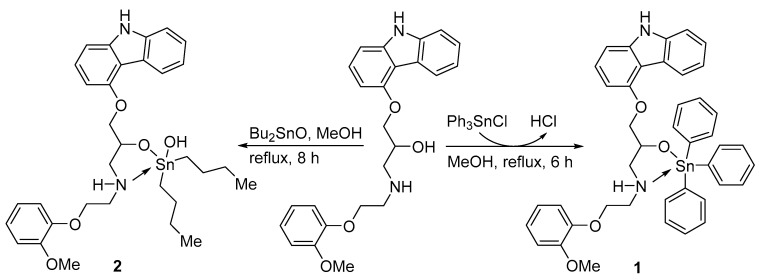
Synthesis of **1** and **2**.

**Scheme 2:**
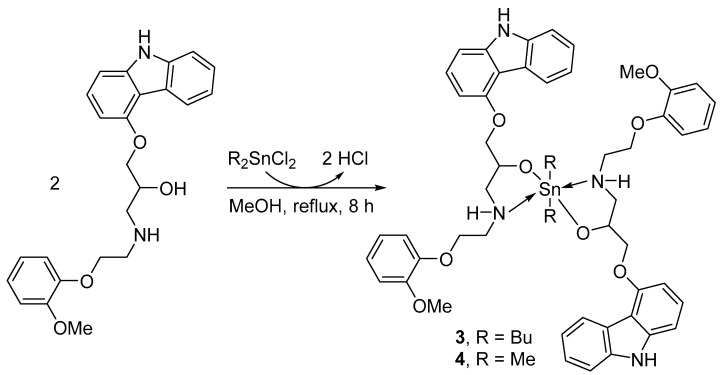
Synthesis of **3** and **4**.

The reaction of carvedilol and either triphenyltin chloride or dibutyltin oxide at a 1:1 ratio in boiling methanol produced the tin complexes **1** or **2**, respectively (Scheme 1), as a white solid. On the other hand, the reaction of carvedilol and appropriate dialkyltin chloride at a 2:1 ratio under similar conditions produced the tin complexes **3** or **4**, respectively (Scheme 2), as a white solid. No base was used in the synthesis of complexes **1–4** which agrees with previously reported procedures [33,34,35]. Table 1 displays the yields, melting points, and elemental analyses (found and calculated %) for **1–4**.

The FTIR spectra of **1–4** exhibited absorption bands resulting from the formation of Sn–C (536–540 cm^–1^), Sn–O (466–474 cm^–1^), and Sn–N (424–447 cm^–1^) bonds which agree with those reported for organotin compounds [35,36]. Furthermore, they exhibited absorption bands for the aromatic and aliphatic N–H bonds at 3340–3410 and 3201–3294 cm^–1^, respectively. The high shift in stretching frequency of aliphatic N–H indicated the occurrence of coordination between tin and nitrogen atoms in carvedilol. Table 2 displays selected FTIR spectral data for **1–4**.

The ^1^H NMR spectra of **1–4** showed the absence of the OH proton that appeared at 5.21 ppm in the ^1^H NMR spectrum of carvedilol, thus confirming the formation of the complexes. ^1^H NMR spectra revealed the presence of two exchangeable singlets that appeared within the 8 ppm and 2 ppm regions because of the aromatic and aliphatic NH protons, respectively (Table 3).

The ^13^C NMR spectra of **1–4** showed all the carbon atoms within the complexes (Table 4). The ^119^Sn NMR spectra of **1** (−136.2 ppm) and **2** (−196.5 ppm) confirmed that the geometry of these complexes is penta-coordinated. Conversely, the geometry of both **3** (−341.8 ppm) and **4** (−213.8 ppm) is hexa-coordinated (Table 4). The ^119^Sn chemical shifts are different for complexes with geometries involving four (+200 to −60 ppm), five (−90 to −190 ppm), and six coordination numbers (−210 to −400 ppm) [37,38,39].

The molar conductivity (10^−3^ M; ethanol) for **1** (2.7 µS/cm), **2** (5.4 µS/cm), **3** (3.5 µS/cm), and **4** (4.8 µS/cm) confirmed that these complexes are non-electrolytic (less than 100 µS/cm) [40,41,42].

### 3.2. PVC Photodegradation Using FTIR Spectroscopy

**Figure 1:**
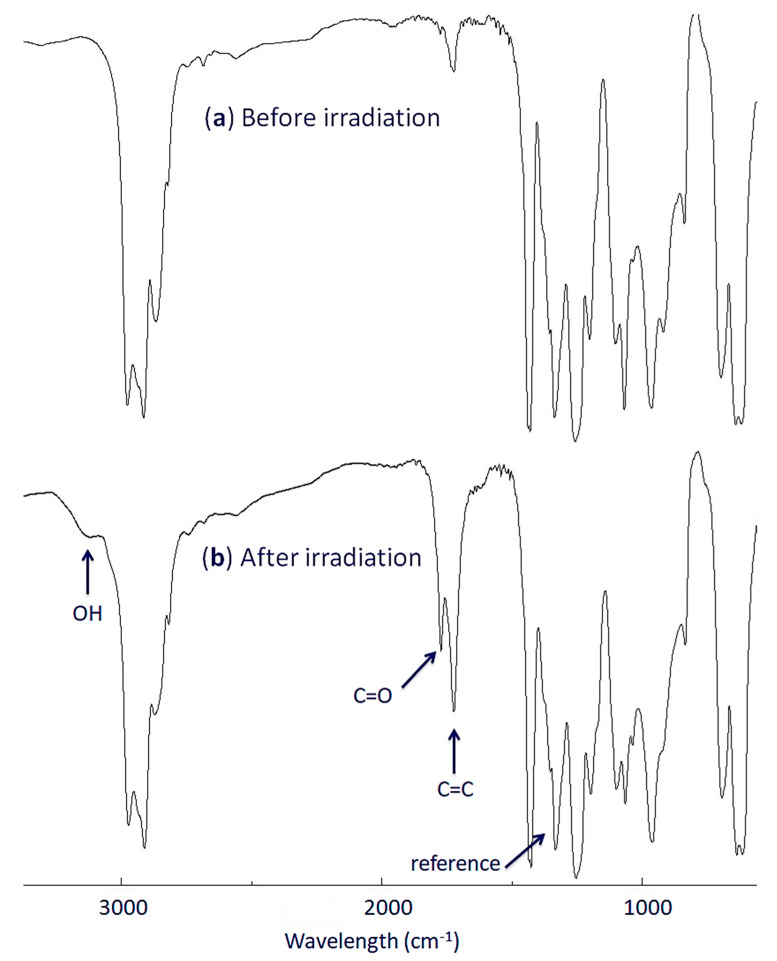
FTIR spectra of PVC: (**a**) before irradiation and (**b**) after irradiation.

**Figure 2:**
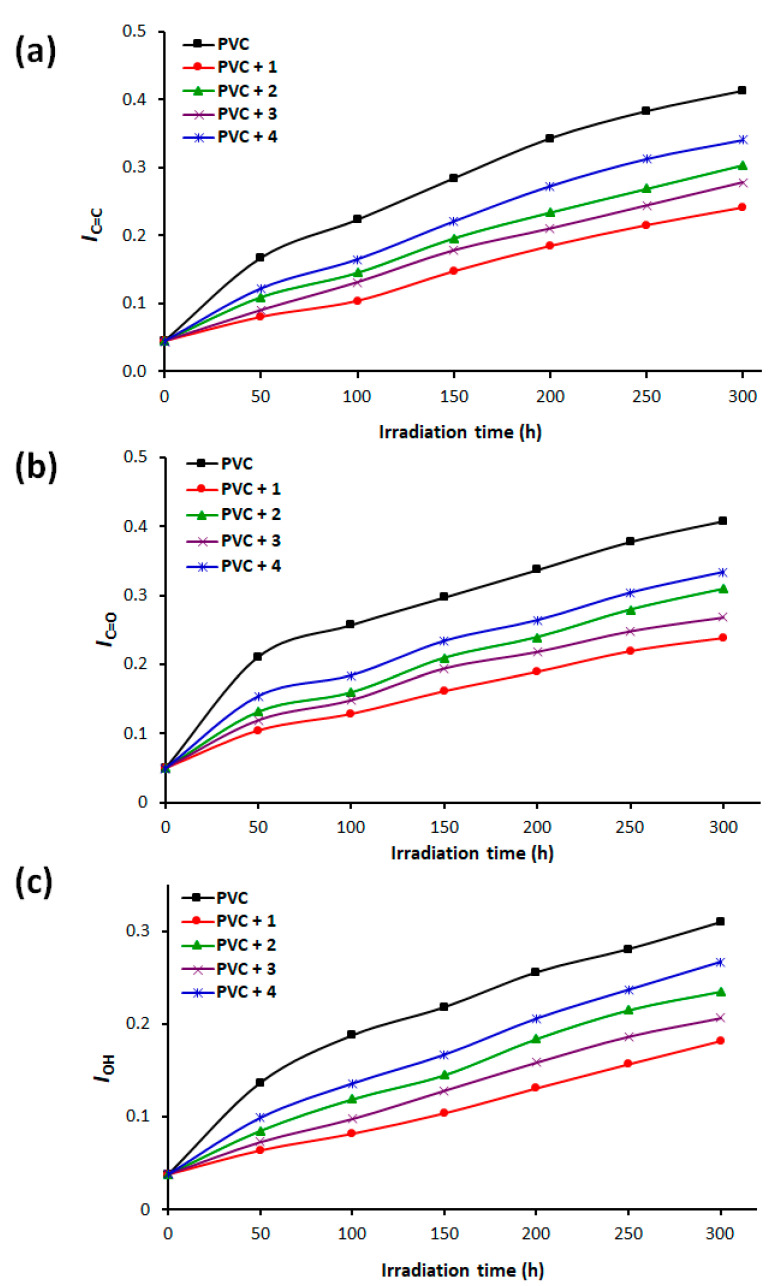
Changes in the functional group indices of Poly(vinyl chloride) (PVC) films (**a**) *I*_C=C_, (**b**) *I*_C=O_, and (**c**) *I*_OH_.

Irradiation causes photooxidation of PVC and promotes the formation of small fragments containing ketones, polyene, and alcohol. The absorbance and intensity of the functional groups corresponding to these fragments can be monitored with FTIR spectroscopy [43,44]. Tin complexes **1–4** were mixed with PVC at a concentration of 0.5% by weight and casted to thin (40 μm) polymeric films. The films were exposed to light with a wavelength of 313 cm^−1^ at 25 °C for up to 300 h. After each 50-h period of irradiation, we recorded the FTIR spectra for the irradiated films. The growth in intensity of C=C (alkenes; 1604 cm^−1^), C=O (ketones; 1772 cm^−1^), and OH (alcohols; 3500 cm^−1^) peaks were monitored and compared with a reference peak (CH_2_; 1328 cm^−1^) [45,46,47]. Figure 1 displays the FTIR spectra of pure PVC film before and after irradiation for 300 hours. It shows that after irradiation, the intensity of C=C, C=O, and OH peaks in the FTIR spectra increased significantly compared to that before irradiation. Equation (1) to calculate the functional group indices (*I*_C=C_, *I*_C=O_, and *I*_OH_). Figure 2 shows the changes in these indices as a function of time (hours).

The *I*_C=C_, *I*_C=O_, and *I*_OH_ of pure PVC film were higher than those of the blends containing additives **1–4**. As the duration of irradiation increased, the functional group indices increased steadily and gradually. The *I*_C=C_ for PVC films, before irradiation was 0.04, and it had increased to 0.41 (blank PVC), 0.24 (PVC/**1** blend), 0.30 (PVC/**2** blend), 0.28 (PVC/**3** blend), and 0.34 (PVC/**4** blend), after irradiation. After irradiation (300 hours), the index in the C=O group had increased from 0.05 to 0.41 (blank PVC), 0.24 (PVC/**1** blend), 0.31 (PVC/**2** blend), 0.27 (PVC/**3** blend), and 0.33 (PVC/**4** blend). Similar increases in the *I*_OH_ (from 0.04 to 0.31) were seen after irradiation. The highly aromatic additive, **1**, was the most effective complex in inhibiting the irradiation-induced growth of functional group indices for PVC; this was followed the additives **3**, **2**, and **4**, in that order. The phenyl rings within **1** act as a good UV absorber and can convert harmful irradiation to harmless heat [14].

### 3.3. PVC Photodegradation Using Weight Loss

**Figure 3:**
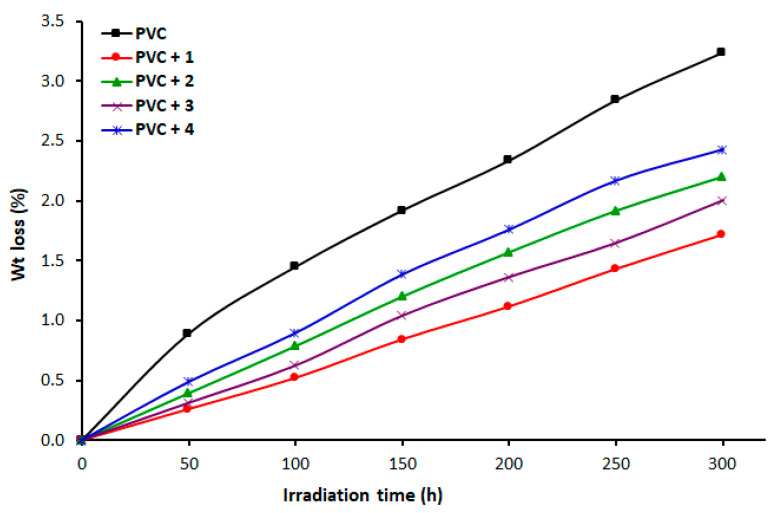
Changes in the weight of PVC films.

Long-term exposure of PVC films to UV irradiation eliminates several by-products, such as volatiles (e.g., HCl) and small fragments with a low molecular weight [48]. Therefore, depending on the duration of exposure, irradiation of PVC causes a loss in the weight of the material [49]. We calculated the PVC weight loss due to irradiation using Equation (2) and plotted it as a function of time (Figure 3). As the duration of irradiation increased, the progression of the weight loss was sharp and steady increased, and it was more significant for the blank PVC material. At the end of the irradiation process (300 h), the percent weight losses of PVC were 3.2%, 1.7%, 2.2%, 2.0%, and 2.4% for pure PVC film, PVC/**1**, PVC/**2**, PVC/**3**, and PVC/**4** blends, respectively. The lowest PVC weight loss was observed when **1** was used as an additive.

### 3.4. Surface Morphology of PVC Films

**Figure 4:**
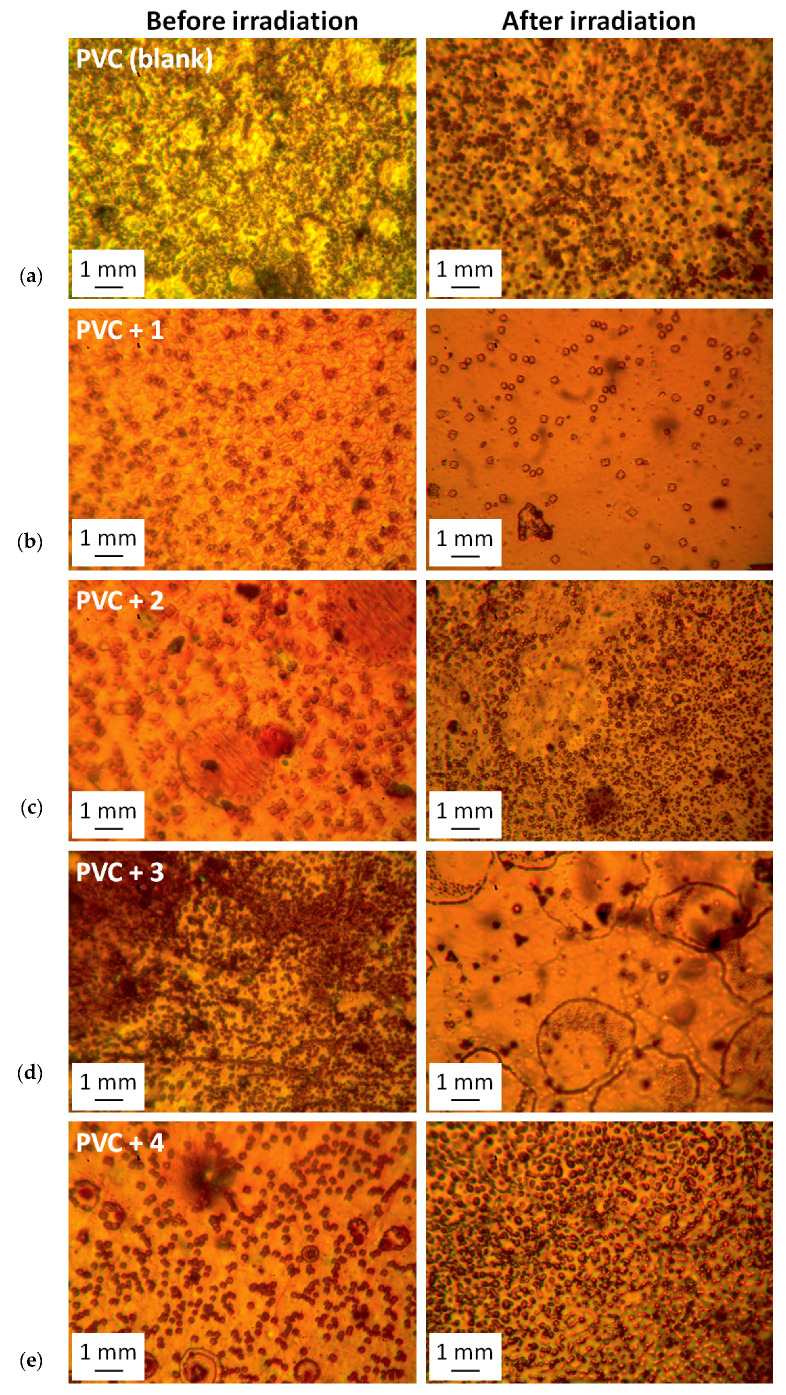
Microscope images (1 mm) of: (**a**) pure PVC, and (**b**) PVC/**1**, (**c**) PVC/**2**, (**d**) PVC/**3**, and (**e**) PVC/**4** blends both before and after irradiation.

Long-term irradiation of PVC causes damage and the appearance of defects and irregularities in the surface of the material [50]. The damage results primarily from dehydrochlorination and chain scission [51]. In the current study, the surface of PVC before irradiation was generally smooth and regular [51]. The microscopic images of PVC films after irradiation (300 h) revealed the formation of grooves, spots, cracks, and a color change (Figure 4). However, for the PVC/tin complex blends, the damage was less significant than that seen in the surface of pure PVC film. The additives reduced the rate of elimination of HCl from PVC and, therefore, improved the photostability of the polymer.

**Figure 5:**
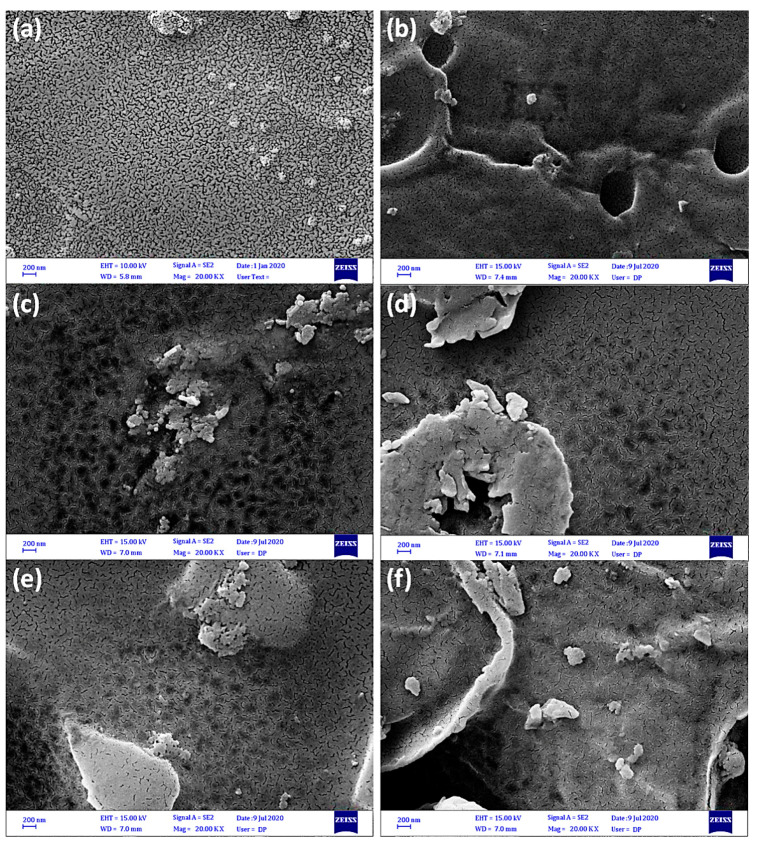
FESEM images of: (**a**) PVC before irradiation, and (**b**) PVC, (**c**) PVC/**1**, (**d**) PVC/**2**, (**e**) PVC/**3**, and (**f**) PVC/**4** films after 300 hours of irradiation.

We inspected the PVC surface morphology further, using a Field Emission Scanning Electron Microscope (FESEM). A FESEM yields high-resolution images that provide detailed information about the homogeneity, shape, cross-sections, and particle sizes of materials [52,53]. FESEM images of non-irradiated PVC generally reveal a smooth and homogenous surface [54]. In this study, the FESEM image (Figure 5a) of non-irradiated PVC (blank) revealed a regular, homogenous, and smooth surface. Moreover, the particles were of similar size and shape. However, the PVC surface of the blends, after irradiation were significantly damaged (Figure 5b–f). Cracks, cavities, spots, irregularities, and variations in the sizes and shapes of particles were more clearly visible on the surface of irradiated pure PVC film (Figure 5b). Such damage and irregularities resulted mainly due to chain cross-linking and elimination of HCl and other small molecules [55]. The findings show that the additives used, particularly additive **1**, significantly reduced the rate of both cross-linking and dehydrochlorination (Figure 5).

**Figure 6:**
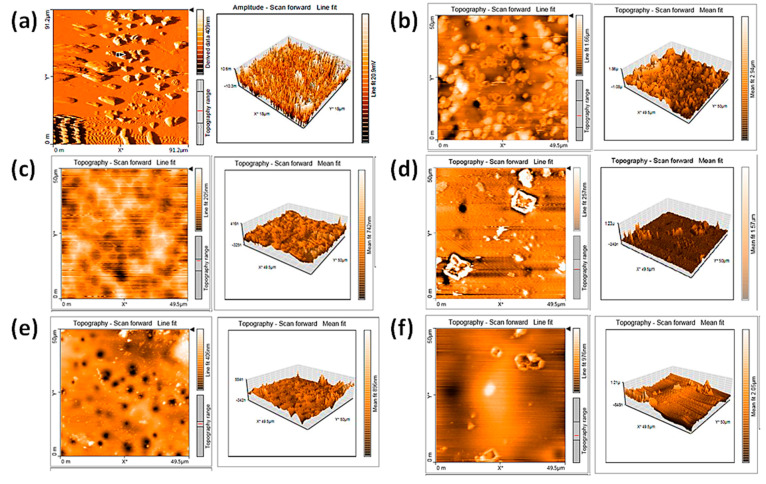
Atomic force microscope (AFM) images of: (**a**) PVC before irradiation, and (**b**) PVC, (**c**) PVC/**1**, (**d**) PVC/**2**, (**e**) PVC/**3**, and (**f**) PVC/**4** films after 300 h of irradiation.

We also used an atomic force microscope (AFM) to provide clear topographic images of the irradiated PVC films. The AFM images indicated that the surfaces of irradiated PVC film were less smooth than they were before irradiation (Figure 6). We analyzed the smoothness of the PVC surface using the roughness factor (*R*q). A high *R*q indicates a low degree of smoothness and considerable irregularity due to dehydrochlorination and bond-breaking of polymeric chains [6]. After irradiation, the surface of PVC/**1** film exhibited a high degree of smoothness (*R*q = 36.3). Conversely, the blank PVC film exhibited a high degree of roughness and irregularities (*R*q = 232.0) after irradiation. It is evident that complex **1** improved the smoothness of the PVC surface 6.4-fold. The *R*q of the irradiated PVC/**2**, PVC/**3**, and PVC/**4** films was 42.2, 44.5, and 137.7, respectively. The additives used provided superior PVC photostabilization to that provided by tin complexes containing 2-(4-Isobutylphenyl)propanoate [56] and naproxen [27], and by Schiff bases containing melamine [15] and triazol-3-thiol [19], and their performance was comparable to that of the tin complex containing furosemide [29].

**Figure 7:**
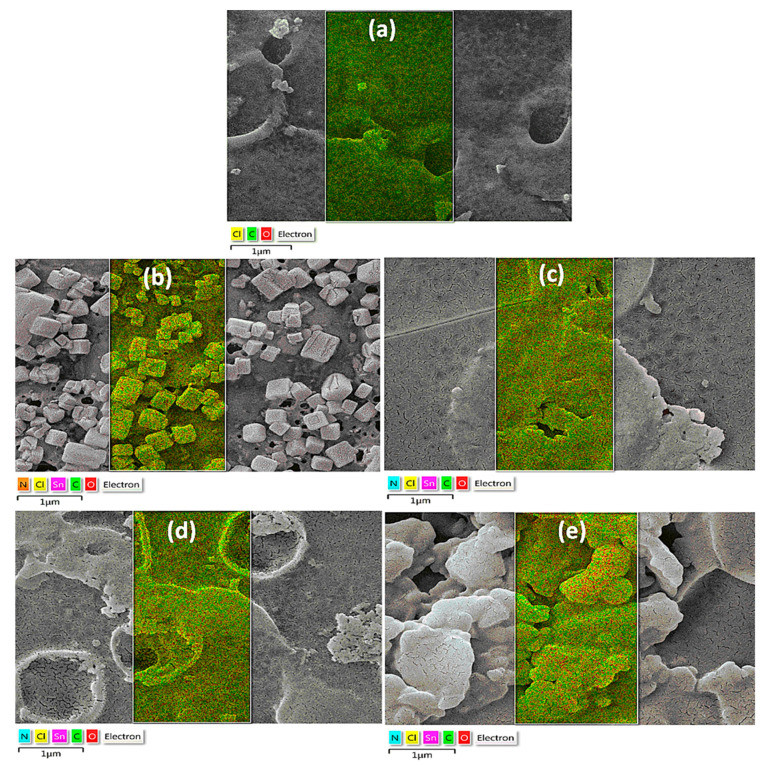
Energy-dispersive X-ray (EDX) mapping of: (**a**) pure PVC film, and (**b**) PVC/**1**, (**c**) PVC/**2**, (**d**) PVC/**3**, and (**e**) PVC/**4** blends after 300 h of irradiation.

The energy-dispersive X-ray (EDX) spectra of PVC blends with tin complexes (Figure 7) revealed new bands corresponding to tin, nitrogen, and oxygen, in addition to carbon and chlorine atoms (high proportion). After irradiation, the carbon content of PVC films increased along with a decrease in chlorine content. The reduction in the chlorine content indicates the elimination of HCl due to photodegradation and photooxidation of PVC. After irradiation, the PVC films containing additives had a higher percentage of chlorine than had with the pure PVC materials. Figure 7 shows the abundance of elements PVC blends after irradiation.

### 3.5. PVC Photostabilization Proposed Mechanisms

**Figure 8:**
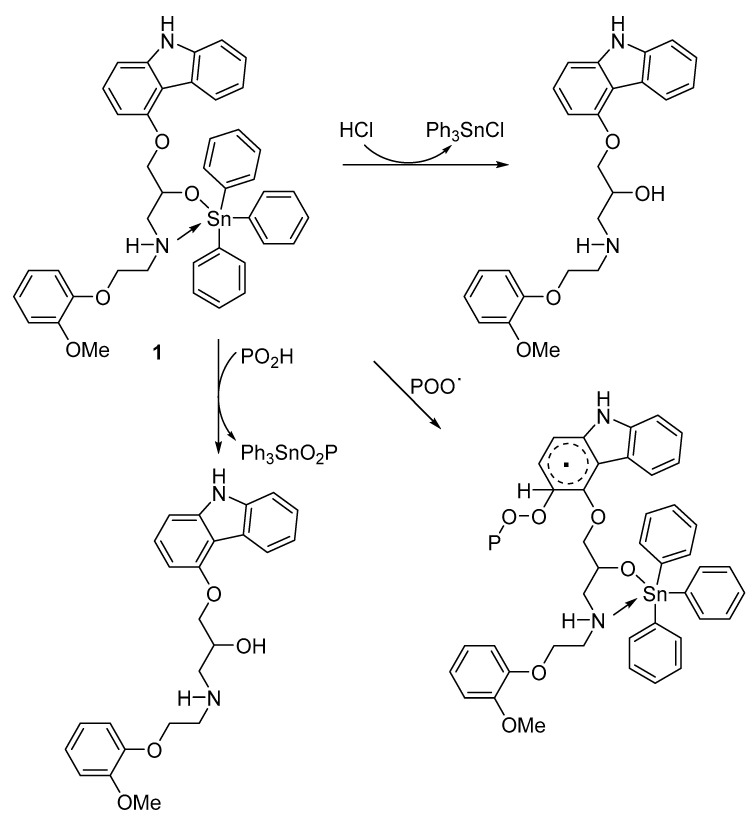
Complex **1** acts as a scavenger of peroxides, radicals, and hydrogen chloride.

Additives **1–4** are shown to be good photostabilizers of PVC, protecting against photodecomposition, and photooxidation of the material, upon exposure to irradiation. The organotin **1** was the most effective additive as photostabilizers of PVC. Additive **1** is highly aromatic, as it contains three phenyl rings in addition to the aryl rings within the skeleton of carvedilol. Additives **2** and **3** contain *n*-butyl groups, whereas additive **4** contains methyl groups. In this study, these additives, particularly additive **1,** acted as UV absorbers, peroxide decomposers, and radical scavengers [14,29,56,57,58,59]. Furthermore, the additives contain the highly acidic tin atom (Lewis acid), they act as excellent scavengers of HCl (Figure 8) [57,60]. The low concentration of **1–4** within the polymeric matrix minimizes any harmful effects of additives when irradiated [60]. Moreover, the coordination between PVC polarized bonds (C–Cl) and electron-rich atoms (e.g., oxygen) in carvedilol stabilize polymeric chains through the transference of energy [29].

## 4. Conclusions

In this study, simple procedures are used to synthesize high yields of several organotin complexes. We analyzed the effect of organotin complexes as inhibitors of PVC photodegradation. Irradiation of thin films of PVC produces destructive changes in the infrared spectra due to the formation of small fragments containing functional groups, loss of weight of the material, and surface defects in the PVC caused by photodegradation. In the presence of tin complexes, such changes were less significant than those that occurred in the blank PVC film. As the concentrations of the additives used were low, their use did not result in changes in either color or homogeneity. The additives used enhanced the photostability of PVC significantly, and the additive with a high degree of aromaticity was particularly effective. Such additives act as absorbers of UV irradiation and scavengers of hydrogen chloride, peroxides, and radicals.

## Figures and Tables

**Table 1:** Yields, melting points, and elemental analyses for **1–4**.

Complex	Yield (%)	MP (°C)	Elemental Analyses (%) Found (Calculated)			
			C	H	N	Sn
**1**	75	121–123	66.86 (66.77)	5.328(5.34)	3.65(3.71)	15.58 (15.71)
**2**	73	110–113	58.56 (58.64)	6.70 (6.77)	4.19 (4.27)	18.01 (18.11)
**3**	71	88–90	64.30 (64.43)	6.51 (6.57)	5.29 (5.37)	12.23 (11.37)
**4**	70	108–111	62.52 (62.57)	5.85 (5.88)	5.71 (5.84)	12.23 (12.37)

**Table 2:** Selected FTIR data for and **1–4**.

Complex	Wave Number (*ν*, cm^–1^)				
	NH (Ar)	NH (Ali)	Sn–C	Sn–O	Sn–N
Carvedilol	3345	3059	—	—	—
**1**	3410	3294	536	466	447
**2**	3340	3201	543	466	439
**3**	3398	3290	540	474	435
**4**	3402	3236	540	474	424

**Table 3:** ^1^H NMR data for and **1–4**.

Complex	ppm, Hz (DMSO-*d_6_*)
Carvedilol	11.28 (s, 1H, NH), 8.29 (d, *J* = 8.1 Hz, 1H, Ar), 7.50 (d, *J* = 8.1 Hz, 1H, Ar), 7.38 (t, *J* = 8.1 Hz, 1H, Ar), 7.35 (d, *J* = 8.1 Hz, 1H, Ar), 7.31 (d, *J* = 8.1 Hz, 1H, Ar), 7.18 (t, *J* = 8.1 Hz, 1H, Ar), 7.12 (d, *J* = 8.1 Hz, 1H, Ar), 6.95–6.71 (m, 4H, Ar), 5.21 (s, exch., 1H, NH), 4.23–4.16 (m, 4H, OH, CH_2_ and CH), 4.03 (t, *J* = 7.1 Hz, 2H, CH_2_), 3.75 (s, 3H, OMe), 2.97 (m, 2H, CH_2_), 2.01 (s, 1H, NH)
**1**	11.28 (s, 1H, NH), 8.28 (d, *J* = 8.0 Hz, 1H, Ar), 7.90–7.82 (m, 5H, Ar), 7.49–7.32 (m, 10H, Ar), 7.28–7.11 (m, 4H, Ar), 6.98–6.86 (m, 5H, Ar), 6.72 (d, *J* = 8.0 Hz, 1H, Ar), 4.25–4.22 (m, 3H, CH_2_ and CH), 4.07–4.02 (m, 2H, CH_2_), 3.75 (s, 3H, OMe), 3.00–2.88 (m, 4H, 2 CH_2_), 2.10 (s, 1H, NH)
**2**	11.28 (s, 1H, NH), 7.80 (d, *J* = 8.0 Hz, 1H, Ar), 7.00 (d, *J* = 8.0 Hz, 1H, Ar), 6.87–7.82 (m, 2H, Ar), 6.69–6.62 (m, 2H, Ar), 6.52–6.40 (m, 4H, Ar), 6.25 (d, *J* = 8.0 Hz, 1H, Ar), 4.73 (s, 1H, OH), 3.77–3.60 (m, 7H, 3 CH_2_ and CH), 3.24–3.08 (m, 6H, 3 CH_2_), 3.30 (s, 3H, OMe), 2.91 (m, 4H, 2 CH_2_), 2.11 (s, 1H, NH), 1.35 (m, 4H, 2 CH_2_), 0.98 (t, *J* = 7.5 Hz, 6H, 2 Me)
**3**	11.36 (s, 2H, 2 NH), 8.26 (d, *J* = 8.0 Hz, 2H, Ar), 7.49 (d, *J* = 8.0 Hz, 2H, Ar), 7.38–7.31 (m, 4H, Ar), 7.15–7.06 (m, 4H, Ar), 7.01–6.93 (m, 8H, Ar), 6.73 (d, *J* = 8.0 Hz, 2H, Ar), 4.35–4.23 (m, 6H, 2 CH_2_ and 2 CH), 3.75 (s, 6H, 2 OMe), 3.28–3.10 (m, 4H, 2 CH_2_), 2.10 (s, 2H, 2 NH), 1.79 (m, 8H, 4 CH_2_), 1.70 (m, 4H, 2 CH_2_), 1.57–1.53 (m, 4H, 2 CH_2_), 1.33 (t, *J* = 7.6 Hz, 4H, 2 CH_2_), 0.87 (t, *J* = 7.6 Hz, 6H, 2 Me)
**4**	11.37 (s, 2H, 2 NH), 8.27 (d, *J* = 8.1 Hz, 2H, Ar), 7.50 (d, *J* = 8.1 Hz, 2H, Ar), 7.39–7.31 (m, 6H, Ar), 7.16–7.12 (m, 4H, Ar), 7.03–6.89 (m, 6H, Ar), 6.74 (d, *J* = 8.1 Hz, 2H, Ar), 4.41–4.15 (m, 6H, 2 CH_2_ and 2 CH), 3.83–3.74 (m, 6H, 3 CH_2_), 3.59 (s, 6H, 2 OMe), 3.32–3.19 (m, 6H, 3 CH_2_), 2.32 (s, 2H, 2 NH), 0.84 (s, 6H, 2 Me)

**Table 4:** ^13^C and ^119^Sn NMR data for carvedilol and **1–4**.

Complex	NMR (ppm, Hz, DMSO-*d_6_*)	
	^13^C	^119^Sn
Carvedilol	156.0, 149.3, 148.2, 141.2, 139.0, 126.5, 124.5, 122.4, 121.8, 121.0, 120.7, 118.6, 113.8, 112.3, 111.7, 110.3, 103.8, 100.5, 70.5, 68.5, 55.5, 52.6, 48.6	—
**1**	155.0, 149.3, 148.1, 141.2, 139.0, 136.2, 135.8, 129.0, 128.1, 126.5, 124.5, 122.5, 121.8, 121.1, 120.7, 118.6, 113.9, 112.3, 111.6, 110.3, 103.9, 100.5, 70.5, 68.4, 68.3, 55.5, 52.5, 48.8	−136.2
**2**	154.5, 148.8, 147.7, 140.7, 138.5, 126.0, 124.0, 122.0, 121.3, 120.6, 120.3, 118.1, 113.4, 111.9, 111.2, 109.9, 104.4, 100.0, 70.0, 68.1, 68.0, 55.0, 52.2, 48.1, 32.0, 27.5, 26.3, 13.5	−169.5
**3**	155.8, 149.0, 147.6, 141.2, 139.0, 126.5, 124.5, 122.5, 121.7, 120.7, 120.5, 118.6, 114.4, 112.3, 111.6, 110.4, 104.0, 100.5, 70.2, 68.2, 68.0, 55.4, 51.5, 47.8, 39.2, 26.7, 25.8, 13.7	−341.8
**4**	154.8, 149.2, 148.0, 141.2, 139.0, 126.5, 124.6, 122.5, 121.7, 120.7, 120.5, 118.6, 114.8, 112.3, 111.6, 110.4, 104.1, 100.5, 70.1, 66.3, 66.2, 55.4, 51.2, 46.6, 6.7	−213.8
